# Fatal Case of a Child Harboring *Enterobius vermicularis*

**DOI:** 10.3390/healthcare11060917

**Published:** 2023-03-22

**Authors:** Samia T. Al-Shouli, Mazin Barry, Khalifa Binkhamis, Nourah AlHogail, Nouf Omar Alafaleq, Osman Adamu Dufailu, Khaldoon Aljerian

**Affiliations:** 1 Immunology Unit, Pathology Department, College of Medicine, King Saud University, Riyadh 12372, Saudi Arabia; 2 Infectious Disease Unit, Department of Medicine, College of Medicine, King Saud University, Riyadh 12372, Saudi Arabia; 3 Medical Microbiology Unit, Department of Pathology, College of Medicine, King Saud University, Riyadh 12372, Saudi Arabia; 4 College of Medicine, King Saud University, Riyadh 12372, Saudi Arabia; 5 Department of Biochemistry, College of Science, King Saud University, Riyadh 12372, Saudi Arabia; 6 Faculty of Engineering and Science, University of Greenwich, Central Avenue, Chatham Maritime, Kent ME4 4TB, UK; 7 Forensic Medicine Unit, Department of Pathology, College of Medicine, King Saud University, Riyadh 12372, Saudi Arabia

**Keywords:** pinworms, enterobiasis, *Enterobius vermicularis*, undiagnosed abdominal pain

## Abstract

*Enterobius vermicularis* is a threadlike parasite also known as “pinworms”. It is the most common helminth infection, affecting the gastrointestinal tracts of children worldwide, although it seldom causes any fatalities. *Enterobius vermicularis* infections are usually asymptomatic and may only cause anal pruritis, with occasional reported cases of ectopic migration into the appendix or the female genital tract by adult pinworms. Here, we report a case of a 15-year-old girl who presented to the emergency department with high-grade fever, vomiting, and vague abdominal pain for three days. She was diagnosed with acute abdominal pain and underwent emergency ileocecectomy, but died the following day. Pathological examination of ileocecal junction showed intraluminal and intramural *Enterobius vermicularis*, which were attributed as the cause of her death in the absence of any other pathologies. Death due to *Enterobius vermicularis* is rare; this case calls for clinicians to be vigilant in exploring *Enterobius vermicularis* infections in patients with undiagnosed acute abdominal pain, since it could be a potential cause of death.

## 1. Introduction

*Enterobius vermicularis*, also known as “pinworms” is considered the most common helminth infection (although being the least pathogenic), affecting the gastrointestinal tract of children worldwide [1]. It accounts for 4–28% of such infections [2,3] and has been well recognized for hundreds of years. In 1634, Fabricus Hildanus described appendicitis pinworms for the first time [4]. There are few case reports of pinworms that show coincidentally in surgical or histologic autopsy specimens, particularly in the appendix. The eggs of these pinworms spread by the fecal–oral route [5,6,7]. Pinworms live and reproduce in various parts of the body, including the ileum, caecum, colon, and appendix. The nematode female migrates to the anus, releases its eggs, and is usually perceived by the host at night [2]. The classical presentation of this infection includes perianal, perineal, or vulvar irritations with secondary sleep disturbance, restlessness, and appetite loss. Since the eggs are deposited outside the intestine, conventional stool microscopy is not a helpful diagnostic tool; however, infection is easily identified using the Graham’s scotch tape method. There have been some extremely rare cases of infection of *Enterobius vermicularis* in the urinary tract, kidney, biliary tree, fallopian tube, and eye (Table 1) [5,8,9,10,11,12,13,14]. However, the vast majority of infected patients remain asymptomatic. Other cases of *Enterobius vermicularis* associated abdominal pain and/or appendicitis have been reported [15,16,17,18]. Deaths attributed directly to this parasite are rarely reported and appear to be diagnosed after autopsy [19]. Recent publications have reported rare complications associated with *Enterobius vermicularis*. Ectopic intramural eggs of *Enterobius vermicularis* were reported to be present in the appendix [20]. In addition, a case report by Harumatsu et al. (2022) observed inflammation and appendicitis with *Enterobius vermicularis* infestation. Blood sample examination results revealed 0.1% eosinophils. Interestingly, neither eosinophil infiltration or granulation, nor obstruction of the appendiceal lumen by *Enterobius vermicularis*, were observed [21]. Here, we report a rare mortality of a young girl in which the cause of death may be attributed to *Enterobius vermicularis* infection.

## 2. Case Description

A 15-year-old girl presented to a tertiary care hospital’s emergency department in Riyadh, Saudi Arabia with a high grade fever, vomiting, and vague abdominal pain for 3 days. She was not known to have had any prior relevant medical history. Furthermore, she had no perianal pruritus, rectal bleeding, chronic diarrhea, or weight loss. None of her household family members had any unusual related symptoms. She was assessed in the same emergency department on two separate visits and was discharged upon the administration of analgesia and antiemetics on both occasions. She returned a third time due to her worsening abdominal pain. On physical examination, she looked unwell, with a temperature of 38.9 °C, pulse rate of 122 bpm, blood pressure of 90/72 mmHg, and respiratory rate of 22 bpm. Abdominal examination showed generalized abdominal tenderness and guarding with the absence of bowel movement.

Laboratory tests revealed a low total white blood cell count of 1.1 × 10^9^/L, and an eosinophils count of 0.1 × 10^9^/L. Other cell counts were normal (Table 2).

Three sets of blood cultures at five days showed no bacterial growth. Her HIV serology was nonreactive. Abdominal X-ray showed mild distended splenic flexure, with normal bowel loop gas distribution, but no free air or air fluid level. While being assessed in the emergency department for a CT scan, her blood pressure dropped to 70/55 mmHg and her abdomen showed progressive distention. She was later taken to the operative room where an emergency exploratory laparotomy was performed. This revealed a large necrotic patch over the caecum. An ileocecectomy was performed, and the patient was put on piperacillin/tazobactam intravenously perioperatively and continued post-operatively. On Day 1 post-surgery, her condition deteriorated further with continued high-grade fever and a further drop in her blood pressure, despite vasopressors. The patient eventually died on the same day.

**Figure 1:**
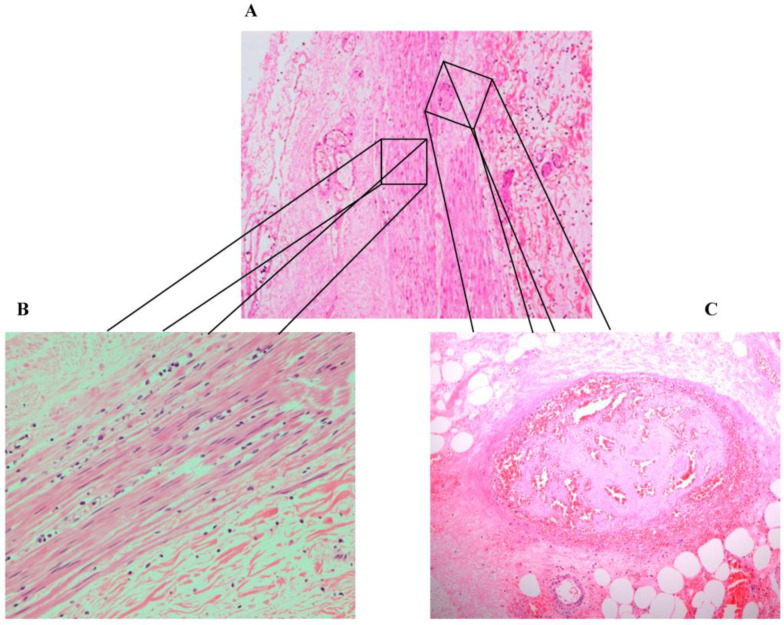
(**A**) Full thick inflammation and necrosis (hematoxylin and eosin stain (H&E), magnification 200×). (**B**) Inflammation. (**C**) Necrosis and thrombosis (hematoxylin and eosin stain (H&E, magnification 200×).

**Figure 2:**
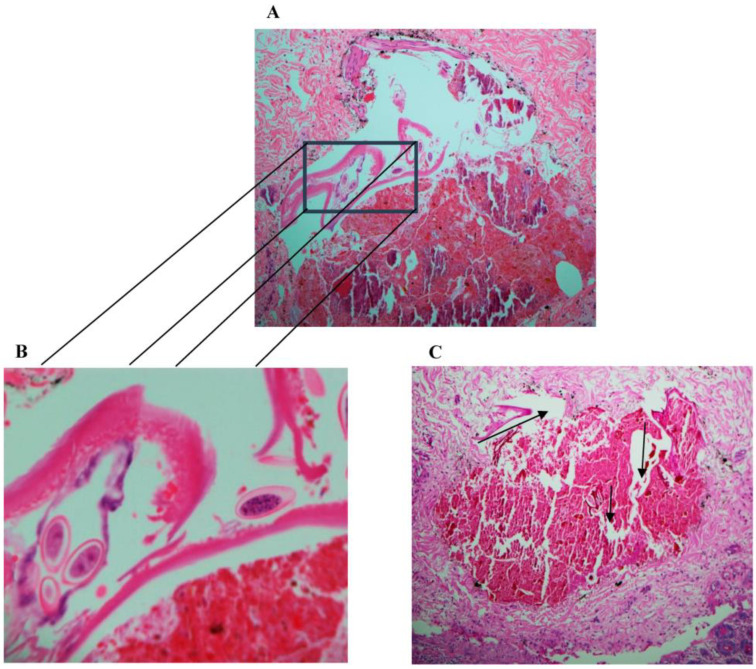
(**A**) Periodic acid–Schiff (PAS), magnification 40×. (**B**) Hematoxylin and eosin stain (H&E), magnification 400×, of *Enterobius vermicularis* worms with eggs. (**C**) Periodic acid–Schiff (PAS), magnification 200×, showing worm embedded in the muscle wall with surrounding necrosis (arrows).

**Figure 3:**
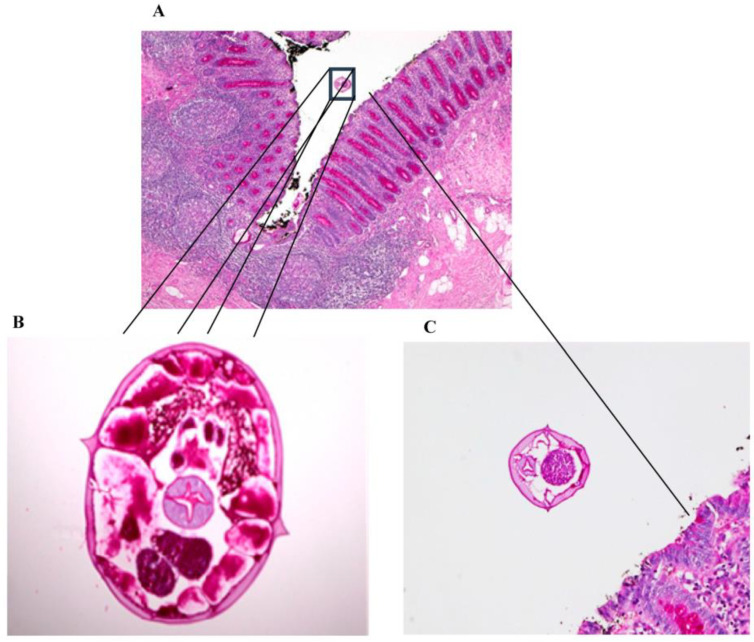
(**A**) Periodic acid–Schiff (PAS), magnification 40×. (**B**) Periodic acid–Schiff (PAS), magnification 200×, of female *Enterobius vermicularis* worms with eggs. (**C**) Periodic acid–Schiff (PAS), magnification 200×, showing worm in the lumen.

**Figure 4:**
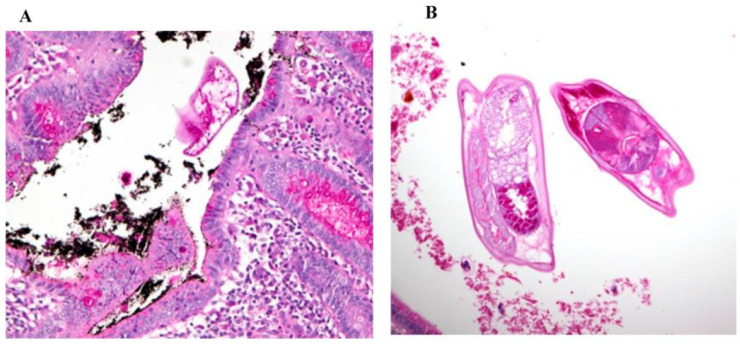
(**A**) Periodic acid–Schiff (PAS), magnification 200×. (**B**) Periodic acid–Schiff (PAS), magnification 200×, showing the presence of elongated worms with slender pointed tails, which is consistent with the morphological characteristics of *Enterobius vermicularis* worms.

At the pathology laboratory, surgical specimens collected before death, including the caecum and part of the ileum, were examined grossly and microscopically. Grossly, the appendix measured 7.5 × 1 cm. Its outer surface was tan pink, unremarkable, and surrounded by the mesoappendix part, which measured 0.5 cm. The serial sectioning showed fecalith material in the lumen. The ileum measured 6 × 6 cm in circumference. Upon opening of the ileum, the mucosa was found to be nodular, with nodules ranging in size between 0.1 and 0.5 cm. The caecum measured 12 × 8 cm in circumference. The outer surface showed scattered white patches, with the largest measuring 2 cm in maximum diameter. Upon opening of the caecum, the mucosal was focally flat and hemorrhagic foci were seen. Microscopically, the appendix was not inflamed and showed viable tissue. However, the caecum and ileocecal junction revealed patchy transmural infarction, vasculitis and vascular thrombosis (Figure 1), and intraluminal and intramural *Enterobius vermicularis* eggs (Figure 2, Figure 3 and Figure 4).

## 3. Discussion

*Enterobius vermicularis* infection is often asymptomatic or presents mild symptoms such as vomiting and perianal pruritus. This case reports the death of a 15-year-old girl who presented with abdominal pain with no perianal pruritus, and no family members with symptoms suggestive of infection. The case was evaluated and attributed to *Enterobius vermicularis* following the pathological examination of surgical specimens. The histopathological findings showed that there were intraluminal and intramural *Enterobius vermicularis* eggs in the ileocecal segment. *Enterobius vermicularis* has been associated with appendicitis, with young girls being the most vulnerable group, although appendicitis by pinworms is still debatable [17,18]. In this young girl, the negative blood cultures and lack of other pathologies makes the cause of death attributable to pinworm.

This is consistent with another report of death attributed to enterobiasis in which the diagnosis was only made after a woman’s autopsy disclosed the presence of *Enterobius vermicularis* localized in the duodenum and proximal ileum with intestinal bleeding [19]. *Enterobius vermicularis* infections can occur asymptomatically among one third of infected people, which makes early diagnosis and intervention challenging. This was the case of the patient in the current study, who did not present with any of the cardinal clinical presentations of *Enterobius vermicularis*, such as perianal pruritus. This naturally presents a challenge to medical teams, as presumptive diagnosis of *Enterobius vermicularis* could not be made without classical symptoms of the disease being presented. Symptoms of perianal irritations could have at least led to perianal swabs being taken in the early morning for microscopy, as stool samples are not ideal because the eggs are deposited outside the large intestines. *Enterobius vermicularis* eggs can be diagnosed with the cellophane tape strategy on perianal skin in the early morning [4].

The severe and worsening abdominal pain experienced by the patient, in the absence of any other relevant medical history, is consistent with other patients in which eosinophilic colitis has been associated with larvae of *Enterobius vermicularis* [22]. Although the low eosinophil level in the blood of the 15-year-old girl was of concern, a recent case report by Harumatsu et al. (2022) reported a similar observation, with eosinophil accounting for 0.1% [21]. Pinworm infections can cause eosinophilic enterocolitis, appendicitis, intestinal obstruction, intestinal perforation, hepatic infection, urinary tract infection, sialadenitis, enterocolitis, eosinophilic ileocolitis, pelvic inflammatory disease, perianal abscesses, and perianal granulomas, and can mimic inflammatory bowel diseases [23]. While in this case the appendix was not inflamed, Souza et al. (2021) reported that *Enterobius* accounts for 68.4% of inflammation [24]. However, intraluminal and intramural *Enterobius vermicularis* eggs were observed in the current case. To the best of our knowledge, intramural *Enterobius vermicularis* eggs have rarely been reported, but recently Mendos et al. (2022) reported a case of ectopic intramural *Enterobius vermicularis* eggs [20]. Furthermore, this current case showcased patchy transmural infarction, vasculitis, and vascular thrombosis in the caecum and ileocecal junction. This shows the complexity of the pathophysiology of *Enterobius vermicularis* infestation, which calls for the need for continues vigilance and surveillance. A cross sectional study conducted on 200 patients who had an appendectomy showed that 30 of 200 appendices (15.0 %) had *Enterobius vermicularis* in histopathological examination; this was found to be more common in females compared to males [25].

Our hypotheses for the probable cause of death is that peripheral blood eosinophilia caused by the parasitic infection promoted a hypercoagulable state that may have resulted in thrombosis leading to ischemia, bowel infarction, and death, which is in line with previous reports with other parasitic infestations [26]. Although many studies have found an association between the presence of thrombosis in different body sites with different parasitic infections [27,28,29,30,31,32,33,34,35,36,37], none have directly linked thrombosis with the *Enterobius vermicularis* infection.

Our case provided limited information since the patient died within hours of admission, which impeded the ability to investigate and check the presence of eosinophilia. In addition, no differential diagnosis was provided, since the patient was rushed to the operating room for an emergency exploratory laparotomy. Moreover, post-mortem examination was not performed because there is no clinical post-mortem examination service available at the hospital, and the hospital does not have a policy on referring autopsies to any other hospital that provides them.

It has been suggested that, owing to the difficulty of diagnosing *Enterobius vermicularis* in the absence of classical symptoms, intestinal parasitosis should be considered in the differential diagnosis of clinical syndromes like gastrointestinal hemorrhages [19]. Could serological intervention help with early detection of *Enterobius vermicularis* in the absence of clinical symptoms? A type-2 oriented immune response has been suggested to be elicited against *Enterobius vermicularis* with potential for balancing activation of the immune system [38]. In spite of this, serological methods are of no diagnostic relevance regarding this parasite [3]. Moreover, since the parasite seldom causes invasiveness, neither blood eosinophilia nor elevated immunoglobulin E levels are generally expected [3]. Moreover, serologic tests for diagnosing pinworm infections are not currently available.

*Enterobius vermicularis* can be primarily controlled by taking daily showers, continued handwashing, and fingernails cutting. A single dose of Mebendazole or Albendazole is effective in reducing the risk of reinfection and complications [3].

## 4. Conclusions

Here we report intraluminal and intramural *Enterobius vermicularis* eggs in a 15-year-old female patient, who unfortunately died. All lifesaving measures to try to save her life were performed; however, it was too late due to gangrene of the bowel. Since the bowel was already gangrenous, all the air in the bowel distended the abdomen. Gangrene from vascular thrombosis and vasculitis led to shock and death. Cases have been reported showing the diversity of the pathophysiology and mode of infestation of *Enterobius vermicularis*, which may cause serious complications, with significant morbidity and mortality, and patients may present with atypical signs and symptoms. Therefore, clinicians should not rule out pinworm infestation in patients with abdominal pain or suspected appendicitis. Our ability to differentially diagnose *Enterobius vermicularis* in the absence of its typical clinical presentations will prevent such fatalities. Unusual parasitic causes such as *Enterobius vermicularis* should be considered in patients with undiagnosed acute abdominal pain by clinicians and pathologists. It is also worth looking into implementing the use of molecular diagnostic tools, such as RNA detection, in these cases.

## Figures and Tables

**Table 1:** Clinical cases described in the text. All cases did not report any travel history.

Location of *Enterobius vermicularis* Infection	Age, Gender, Country of Origin	Therapy	Outcome	Ref
Urinary tract	65-year-old male from Greece	Mebendazole 100 mgs for 3 days followed by two more courses with 3 week time interval.	Complete resolution of symptoms.	[8]
Bladder	54-year-old female from Brazil	Mebendazole 200 mgs for 3 days.	Complete resolution of the voiding symptoms and normalization of urinalysis.	[9]
Kidney	51-year-old female from France	Nephrectomy and amoxicillin; gentamicin.	Complete resolution of symptoms.	[10]
Biliary tree	73-year-old female from UK	Laparoscopic cholecystectomy with intra-operative cholangiogram	Recovered after surgery.	[11]
Fallopian tube	23-year-old female from Malaysia	Laparotomy with a right salpingo-oophorectomy. Albendazole for a week upon discharge.	Recovered after surgery.	[12]
Pelvis	11-year-old female from Greece	Laparotomy was performed. Intravenous cefoxitin, amikacin, and metronidazole postoperatively.	Recovered after Surgery.	[13]
Nares and ocular orbit	14-year-old female from the United States	Mebendazole 300 mg twice daily for 3 days.	Complete resolution of symptoms.	[14]

**Table 2:** Patient blood cell count.

Blood Cell Count	Patient’s Results	Reference Range
Total white blood cell	1.1 × 10^9^/L	4.5–11.0 × 10^9^/L
Lymphocytes		1.5–4.0 × 10^9^/L
Neutrophils		2–7.5 × 10^9^/L
Eosinophils	0.1 × 10^9^/L	0.0–0.4 × 10^9^/L
Monocytes		0.2–1.0 × 10^9^/L
Basophils		0.0–0.5 × 10^9^/L

## Data Availability

Data is available upon request.

## References

[1] Neva F.A., Brown H.W. (1994). Basic Clinical Parasitology.

[2] Lamps L.W. (2010). Surgical Pathology of the Gastrointestinal System: Bacterial, Fungal, Viral, and Parasitic Infections.

[3] Wendt S., Trawinski H., Schubert S., Rodloff A.C., Mössner J., Lübbert C. (2019). The Diagnosis and Treatment of Pinworm Infection. Dtsch. Arztebl. Int..

[4] Stĕrba J., Vlcek M., Noll P., Vorel F. (1985). Contribution to the question of relationships between *Enterobius vermicularis* (L.) and inflammatory processes in the appendix. Folia Parasitol..

[5] Kucik C.J., Martin G.L., Sortor B.V. (2004). Common intestinal parasites. Am. Fam. Physician.

[6] Arkoulis N., Zerbinis H., Simatos G., Nisiotis A. (2012). Enterobius vermicularis (pinworm) infection of the liver mimicking malignancy: Presentation of a new case and review of current literature. Int. J. Surg. Case Rep..

[7] Isik B., Yilmaz M., Karadag N., Kahraman L., Sogutlu G., Yilmaz S., Kirimlioglu V. (2007). Appendiceal Enterobius vermicularis infestation in adults. Int. Surg..

[8] Zahariou A., Karamouti M., Papaioannou P. (2007). Enterobius vermicularis in the male urinary tract: A case report. J. Med. Case Rep..

[9] Sammour Z.M., Gomes C.M., Tome A.L., Bruschini H., Srougi M. (2008). Prolonged irritative voiding symptoms due to Enterobius vermicularis bladder infestation in an adult patient. Braz. J. Infect. Dis. Off. Publ. Braz. Soc. Infect. Dis..

[10] Cateau E., Yacoub M., Tavilien C., Becq-Giraudon B., Rodier M.H. (2010). Enterobius vermicularis in the kidney: An unusual location. J. Med. Microbiol..

[11] Dick L., Hannay J. (2017). *Enterobius vermicularis* presentation during laparoscopic cholecystectomy. J. Surg. Case Rep..

[12] Ngui R., Ravindran S., Ong D.B., Chow T.K., Low K.P., Nureena Z.S., Rajoo Y., Chin Y.T., Amir A., Ahmad A.F. (2014). *Enterobius vermicularis* salpingitis seen in the setting of ectopic pregnancy in a Malaysian patient. J. Clin. Microbiol..

[13] Mentessidou A., Theocharides C., Patoulias I., Panteli C. (2016). *Enterobius vermicularis*-Associated Pelvic Inflammatory Disease in a Child. J. Pediatr. Adolesc. Gynecol..

[14] Babady N.E., Awender E., Geller R., Miller T., Scheetz G., Arguello H., Weisenberg S.A., Pritt B. (2011). *Enterobius vermicularis* in a 14-year-old girl’s eye. J. Clin. Microbiol..

[15] Hammood Z.D., Salih A.M., Mohammed S.H., Kakamad F.H., Salih K.M., Omar D.A., Hassan M.N., Sidiq S.H., Mustafa M.Q., Habibullah I.J. (2019). *Enterobius vermicularis* causing acute appendicitis, a case report with literature review. Int. J. Surg. Case Rep..

[16] Taghipour A., Olfatifar M., Javanmard E., Norouzi M., Mirjalali H., Zali M.R. (2020). The neglected role of *Enterobius vermicularis* in appendicitis: A systematic review and meta-analysis. PLoS ONE.

[17] Efared B., Atsame-Ebang G., Soumana B.M., Tahiri L., Hammas N., El Fatemi H., Chbani L. (2017). Acute suppurative appendicitis associated with *Enterobius vermicularis*: An incidental finding or a causative agent? A case report. BMC Res. Notes.

[18] Rajendran S., Carmody E., Murphy M., Barry B. (2015). *Enterobius* granulomas as a cause of abdominal pain. BMJ Case Rep..

[19] Mansueto G., De Simone M., Ciamarra P., Capasso E., Feola A., Campobasso C.P. (2021). Infections Are a Very Dangerous Affair: Enterobiasis and Death. Healthcare.

[20] Mendos A., Mathison B.A., Pritt B.S., Lamps L.W., Pai S.A. (2022). Intramural Ova of *Enterobius vermicularis* in the Appendix—An Egg-Topic Location!. Int. J. Surg. Pathol..

[21] Harumatsu T., Baba T., Orokawa T., Sunagawa H., Ieiri S. (2022). A rare case of acute appendicitis with *Enterobius vermicularis*. Pediatr. Int..

[22] Liu L.X., Chi J., Upton M.P., Ash L.R. (1995). Eosinophilic colitis associated with larvae of the pinworm *Enterobius vermicularis*. Lancet.

[23] Rajamanickam A., Usmani A., Suri S., Dimov V. (2009). Chronic diarrhea and abdominal pain: Pin the pinworm. J. Hosp. Med..

[24] Sousa J., Hawkins R., Shenoy A., Petroze R.T., Mustafa M.M., Taylor J.A., Larson S.D., Islam S. (2021). *Enterobius vermicularis*-associated appendicitis: A 22-year case series and comprehensive review of the literature. J. Pediatr. Surg..

[25] Hamdona S.M., Lubbad A.M., Al-Hindi A.I. (2016). Histopathological study of *Enterobius vermicularis* among appendicitis patients in Gaza strip, Palestine. J. Parasit. Dis. Off. Organ Indian Soc. Parasitol..

[26] Ames P.R., Aloj G., Gentile F. (2011). Eosinophilia and thrombosis in parasitic diseases: An overview. Clin. Appl. Thromb. Hemost. Off. J. Int. Acad. Clin. Appl. Thromb. Hemost..

[27] Moxon C.A., Alhamdi Y., Storm J., Toh J.M.H., McGuinness D., Ko J.Y., Murphy G., Lane S., Taylor T.E., Seydel K.B. (2020). Parasite histones are toxic to brain endothelium and link blood barrier breakdown and thrombosis in cerebral malaria. Blood Adv..

[28] Waters M., Krajden S., Kim C., Elsobky R., Lychacz B., Cheung M., Crowther M., Keystone J. (2019). Case Report: Two Cases of Strongyloidiasis Presenting with Thrombotic Events. Am. J. Trop. Med. Hyg..

[29] Nowak S.P., Pielok Ł., Stefaniak J. (2019). Thrombosis of inferior vena cava in the course of advanced alveolar echinococcosis. Pol. Arch. Intern. Med..

[30] Ozsay O., Gungor F., Karaisli S., Kokulu I., Dilek O.N. (2018). Hydatid cyst of the pancreas causing both acute pancreatitis and splenic vein thrombosis. Ann. R. Coll. Surg. Engl..

[31] Kaur J., Gupta A., Wadhwa N. (2017). Hepatic Visceral Larva Migrans Causing Hepatic Venous Thrombosis and Prolonged Fever. Indian Pediatr..

[32] Takeda A., Hayashi S., Teranishi Y., Imoto S., Nakamura H. (2017). Portomesenteric Vein Thrombosis After Excision of Parasitic Peritoneal Myomas. J. Minim. Invasive Gynecol..

[33] Mosli M.H., Chan W.W., Morava-Protzner I., Kuhn S.M. (2016). Schistosomiasis Presenting as a Case of Acute Appendicitis with Chronic Mesenteric Thrombosis. Can. J. Infect. Dis. Med. Microbiol..

[34] Leite L.A.C., de Cássia dos Santos Ferreira R., Hatzlhofer B.L.D., Correia M.C.B., Bandeira Â.P., Owen J.S., Lima V., Domingues A.L.C., Lopes E. (2016). Portal vein thrombosis associated with protein C deficiency and elevated Factor VIII in hepatosplenic schistosomiasis. Blood Coagul. Fibrinolysis Int. J. Haemost. Thromb..

[35] Abo-Salem E.S., Ramadan M.M. (2015). A huge thrombosed pulmonary artery aneurysm without pulmonary hypertension in a patient with hepatosplenic schistosomiasis. Am. J. Case Rep..

[36] Singla N., Gupta M., Singh R., Kumar A. (2014). Atypical neurological manifestations of malaria. BMJ Case Rep..

[37] Olveda D.U., Olveda R.M., Montes C.J., Chy D., Abellera J.M.B., Cuajunco D., Lam A.K., McManus D.P., Li Y., Ross A.G.P. (2014). Clinical management of advanced schistosomiasis: A case of portal vein thrombosis-induced splenomegaly requiring surgery. BMJ Case Rep..

[38] Patsantara G.G., Piperaki E.T., Tzoumaka-Bakoula C., Kanariou M.G. (2016). Immune responses in children infected with the pinworm *Enterobius vermicularis* in central Greece. J. Helminthol..

